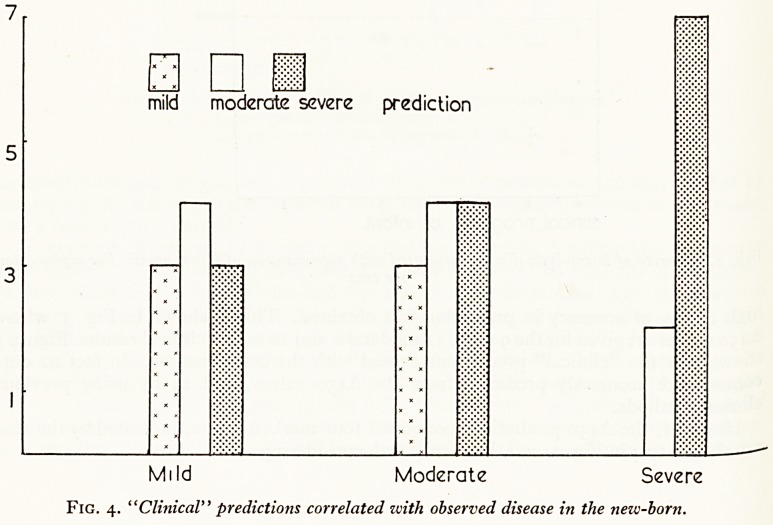# A Review of 33 Liquor Amnii Analyses during Pregnancy Complicated by Rhesus Incompatibility

**Published:** 1965-10

**Authors:** R. A. Branch


### A REVIEW OF 33 LIQUOR AMNII ANALYSES DURING PREGNANCY COMPLICATED BY RHESUS INCOMPATIBILITY*

BY

R. A. BRANCH

In rhesus-negative women who have had previous babies severely affected by rhesus incompatibility, the maternal rhesus antibody titre during a subsequent pregnancy offers only a poor indication of the degree to which the developing foetus will be affected. This has induced workers to look for laboratory tests that can give more reliable information. In an attempt to detect excessive haemolysis in the foetus, Walker (1957) made spectrophotometric measurements of the liquor amnii, and found that the amount of yellow pigment that it contained corresponded closely with the degree of anaemia of the baby, as measured by the haemoglobin content of cord blood.

Liley (1961) in Auckland applied Walker's technique in 101 selected cases. He usually obtained two liquor amnii samples, one at 29 weeks and the other at 31–32 weeks gestation. These were centrifuged, and the optical density was determined over a range of wavelengths. For normal liquor, the graph of optical density against wavelength is nearly a straight line. If there has been excessive haemolysis in the foetus there is an increase of the yellow pigment in the liquor, and a corresponding increase in the optical density around 450 m$\mu$ (Fig. 1). Liley measured the increase above the expected base-line, and referred to it as the "$\Delta$450" value. He found it to be closely and directly related to the degree to which the foetus was affected; and the degree to which the $\Delta$450 was raised allowed the prediction of mild, moderate, and severe affection of the infant. In normal pregnancies there was a small rise in the $\Delta$450 at 28–32 weeks, but this decreased in the later weeks. The raised $\Delta$450 of a mildly affected infant also decreased with increasing gestation, but a rising $\Delta$450 was a definite indication of a severe haemolytic state.

### THE INVESTIGATION

Thirty-three patients have been reviewed from whom liquor amnii samples were sent to the Biochemistry Laboratory at Southmead Hospital. Patients were selected for amniocentesis when other methods of prediction were thought to be of doubtful value. The majority of patients previously had severely affected infants.

Three cases were excluded; one patient moved from the Bristol area to have her baby, the second was delivered of a still-born anencephalic baby in whom no assessment of the haemolytic state was made, and in the third case there was contamination of the liquor amnii sample with blood.

The patients, admitted to hospital as "day cases," had been dealt with as follows: 1–4 mls of 0.5 per cent lignocaine was injected locally into the anterior abdominal wall in the most convenient site as determined by abdominal palpation. A Howard-Jones needle was directed into the amniotic cavity anterior to the flexed body of the foetus to avoid vital organs. 5–10 mls of amniotic fluid were collected in a sterile container and stored in a refrigerator. Later the sample was centrifuged for 10 minutes

* A student project in the Department of Obstetrics and Gynaecology, University of Bristol, abridged for publication.

and optical densities at varying wavelengths from 350 mμ to 700 mμ were measured on an undiluted sample using a Hilgar Unispec Spectrophotometer.

Figure 1 shows an optical density graph of a normal control and of a severely affected sample of liquor; it shows how the Δ450 is obtained by projecting the base-line for each sample. If blood contaminates a sample it can be allowed for, because it causes

FIG. 1. *Optical density of liquor amnii at various wavelengths.*
*Lower curve—normal liquor.*
*Upper curve—liquor in haemolytic disease.*

an absorption peak at 415 mμ, 5 per cent of which is present at 450 mμ, so that by measuring the rise above the expected base-line at 415 mμ a correction can be made, and a true Δ 450 obtained.

In practice, of course, all available data had been used in deciding how to manage each patient. In this study a comparison is made between two methods of prediction (a) one based on the Δ450 alone, and (b) a clinical prediction based on the previous obstetric history, the husband's presumed genotype, and the maternal antibody titre. By each method the prediction of haemolytic disease as "mild", "moderate", or "severe" was compared with the actual findings in the baby after birth, recorded under the same headings. To obtain these ratings in the new-born baby, the cord haemoglobin was considered to be a poor guide; only values below "90 per cent" indicated a severe haemolytic state. The assessment was made on the degree of hyperbilirubinaemia and the necessity for exchange blood transfusions. The classification used was as follows, the letters (a) to (g) referring to Fig. 2:—

*Mild* (a) Not affected.
(b) Mild jaundice; no transfusion.
*Moderate* (c) Moderate jaundice; 1 exchange transfusion after 48 hours.
(d) Moderate jaundice; 1 exchange transfusion within 48 hours.
*Severe* (e) Severe jaundice; more than 1 exchange transfusion.
(f) Neonatal death from haemolytic disease.
(g) Still birth.

Figure 2 shows how the clinical state of the infant was related to the Δ450 of the amniotic fluid. Following Liley (1961) the Δ450 values were devided into three ranges, and it was found that by limiting the "middle range" to between 0·040 and 0·070 a

FIG. 2. *Severity of haemolytic disease correlated with pigmentation of liquor amnii. For explanation see text.*

high degree of accuracy in prediction was obtained. This is shown in Fig. 3, where Δ450 ranges are given for the 9 mild, 11 moderate, and 10 severe clinical results. Figure 4 shows how the "clinical" predictions agreed with the actual results. In fact 22 outcomes were accurately predicted from the Δ450 values, and 14 by using previous clinical methods.

However, the Δ450 predictions contained four marked errors, indicated by the case numbers 1 to 4 in Fig. 2, and these are worth considering.

FIG. 3. "Δ450" *predictions correlated with observed disease in the new-born.*

FIG. 4. "*Clinical*" *predictions correlated with observed disease in the new-born.*

*Case* 1. This patient, with a homozygous husband, had previously had a severely affected infant. During the pregnancy the maternal rhesus antibody titre rose from 1:20/40 to 1:80, but her Δ450 was only 0·035. In spite of the Δ450 a severely affected foetus was correctly predicted with successful premature induction at 36 weeks. This was the only occasion in which a low Δ450 was found with an affected foetus.

*Case* 2. This patient, with a heterozygous husband, had previously had an infant dying from haemolytic disease. The maternal antibody titre remained at 1:80 and the Δ450 was 0·051 indicating a moderately affected baby. Premature induction at 36 weeks produced an unaffected rhesus-negative baby who progressed satisfactorily.

*Case* 3. This patient had a homozygous husband; she had had three normal unaffected pregnancies, then in her fourth her baby died shortly after birth. This was thought to be due to haemolytic disease, but subsequent questioning revealed the primary cause to be foetal asphyxia following an accidental antepartum haemorrhage associated with pre-eclampsia. The rhesus antibody titre remained at 1:80, the Δ450 was 0·056. As a severely affected baby was suspected and maternal health was poor, an elective lower segment caesarean section was performed at 36 weeks. The unaffected rhesus positive infant nearly died from idiopathic respiratory distress syndrome.

*Case* 4. The patient with a heterozygous husband had her first child normally. Her second and third died of hydrops foetalis, her fourth was rhesus-negative. The rhesus antibody titre remained at 1:10 and the Δ450 was 0·095 indicating a severely affected baby. Premature induction at 35½ weeks produced an unaffected rhesus-negative baby who progressed satisfactorily.

### DISCUSSION

The results of this study confirm Liley's report that spectrophotometric analysis of liquor amnii is a sensitive diagnostic aid, but not invariably accurate. It is significant that in this highly selected group of patients, 10 pregnancies previously suspected to be severely affected were able to continue to 38 weeks, when it was thought wisest to induce labour, and only one baby died of haemolytic disease.

In order to reduce the number of wrong predictions, Liley advises that two liquor amnii samples should be taken. This had been done in four of the present cases with inconclusive results. It is debatable whether the risk of amniocentesis is low enough to justify two samples in every case. In this series only one mother had any referable complication. This patient was admitted at 36 weeks with an antepartum haemorrhage two days following an aminocentesis. She spontaneously went into labour and a "Class A" baby severely affected by haemolytic disease was born.

The Δ450 is purely a measure of yellow pigment in the liquor amnii; only a small proportion of this pigment gives a positive diazo reaction for bilirubin; the chemical nature of the remaining pigment is unknown. But as the total pigment appears to be related to haemolytic disease, the simple optical density measurement is more sensitive than the cruder chemical techniques. There is a possibility that the false high readings may be caused by other types of yellow pigment, though no simple chemical tests have yet been devised to prove it. The application of this test in selecting severely affected foetuses for intra-uterine transfusion by means of a needle passed into the foetal peritoneal cavity (Holman and Karnicki, 1964) is promising.

### CONCLUSION AND SUMMARY

Thirty patients have had spectrophotometric analysis of their liquor amnii. The "Δ450" is shown to be a highly sensitive index of the degree to which the foetus is affected. The outcome of 22 cases was accurately predicted, with only four misleading results, and four equivocal results. This compared favourably with previous methods of prediction whereby 14 outcomes were correctly predicted, three were wrongly predicted, and 13 equivocal predictions were made.

It is possible that the accuracy of prediction from analysis of liquor amnii would be improved if two amniocenteses were routinely performed and if further knowledge of the chemical nature of the pigment could be obtained.

*Acknowledgements*

I would like to thank Professor G. G. Lennon, Dr. G. H. Tovey and Dr. J. B. Holton for their help.

### REFERENCES

Holman, C. A. and Karnecki, J. (1964). *Brit. med. J.* **ii**, 597.

Liley, A. W. (1961). *Amer. J. Obstr. Gynec.* **82**, 1359.

McCrostie, H. M. (1964). *Brit. med. J.* **i**, 920.

Walker, A. H. C. (1957). *Brit. med. J.* **ii**, 376.

## Figures and Tables

**Fig. 1. f1:**
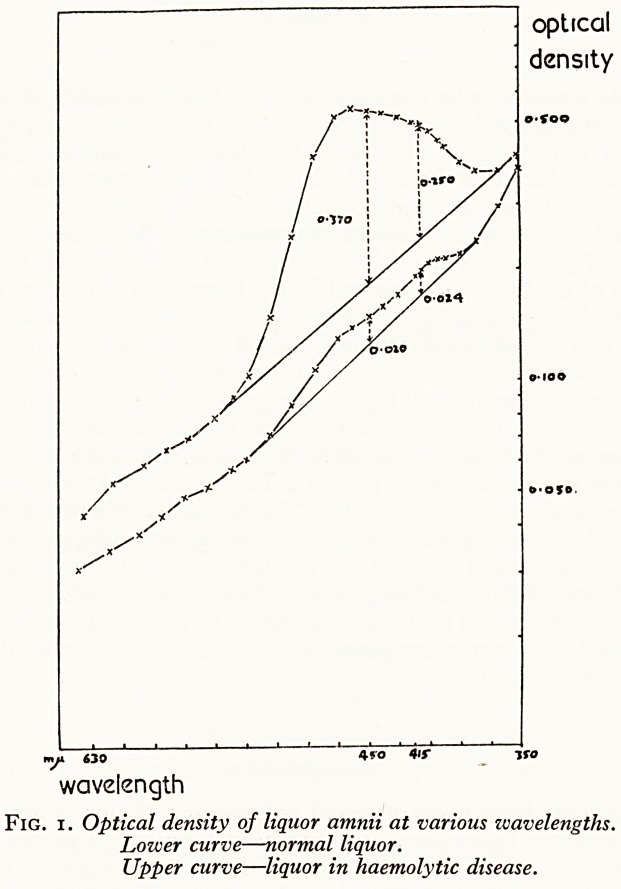


**Fig. 2. f2:**
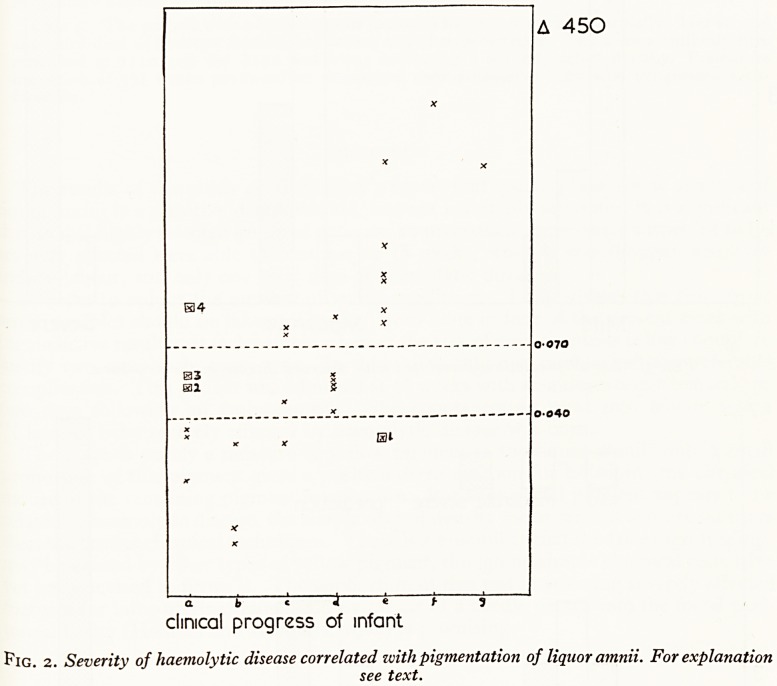


**Fig. 3. f3:**
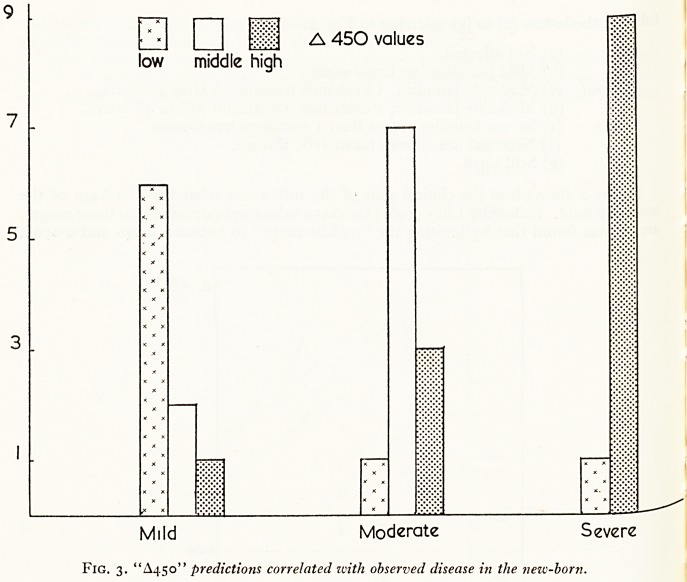


**Fig. 4. f4:**